# Transmission of SARS-CoV-2 and Other Infections at Large Sports Gatherings: A Surprising Gap in Our Knowledge

**DOI:** 10.3389/fmed.2020.00277

**Published:** 2020-05-29

**Authors:** Michele Sassano, Martin McKee, Walter Ricciardi, Stefania Boccia

**Affiliations:** ^1^Department of Life Science and Public Health, Section of Hygiene and Public Health, Università Cattolica del Sacro Cuore, Rome, Italy; ^2^London School of Hygiene and Tropical Medicine, London, United Kingdom; ^3^Department of Woman and Child Health and Public Health - Public Health Area, Fondazione Policlinico Universitario A. Gemelli IRCCS, Rome, Italy

**Keywords:** COVID-19, SARS-CoV-2, sports, gatherings, sporting events, outbreaks, epidemics, pandemics

The first case of locally transmitted SARS-CoV-2 infection in Italy was recorded on February 18, 2020, in Codogno, in the region of Lombardy (1). Since then, the number of cases has increased rapidly, with 225,435 cases (or 0.37% of the population) by 17th May, 31,908 (14.15%) dying and 125,176 (55.53%) recovering (1). Lombardy is the epicenter of the disease in Italy. Accounting for 16.7% of the Italian population, it has had 37.64% of the country's cases, numbering 84,844 or 0.84% of the population (1). Within Lombardy, the province of Bergamo is among the hardest-hit in the country, with 12,443 COVID-19 cases, or 1.12% of the population. During March 2020, it saw an increase of 567.6% in daily deaths compared with the average in March 2015–2019 (Table 1) (1, 2). While the reason that Bergamo was so badly affected remains uncertain, several commentators, including the local mayor, have pointed to a football match.

**Table 1:** Number of cases and deaths due to Covid-19 in Italy, Lombardy, and province of Bergamo in the weeks after Atalanta—Valencia CF football match held on 19th February. (2–5).

	**Three weeks later (March 11, 2020)**		**Six weeks later (March 31, 2020)**		**March 2020**
**Area**	**Cases**	**Deaths (% of cases)**	**Cases**	**Deaths (% of cases)**	**Change in daily deaths compared with the average value on March 2015-2019**
Italy	12,462	827 (6.6%)	105,792	12,428 (11.7%)	+49.4%
Lombardy	7,280	617 (8.5%)	43,208	7,199 (16.7%)	+186.5%
Bergamo	1,815	-	8,803	2,060^*^ (23.4%)	+567.6%

* *Number reported by local media as recognized by authorities*.

The match in question was the UEFA Champions League (UCL) match between Atalanta, Bergamo's sole professional team, and Spain's Valencia CF, held in the San Siro stadium in Milan on 19th February, just before the epidemic took off (Table 1) (6). It was a momentous event for Bergamo, being its first appearance in UCL. Of the 45,792 tickets sold, an estimated 95% (43,500 persons) were bought in Italy, with only 5% (2,500) in Spain. If we assume that the vast majority of Atalanta supporters were from Bergamo city, then one in three of its population attended the match. An event such as this provides many opportunities for mixing, not only in the stadium, but in transport to and from the match and in bars and similar venues before and after it, which can be expected to have been crowded given Atalanta's unprecedented victory. It would have been impossible to have maintained social distancing, even if it had been attempted. Many of those who did not travel to Milan likely congregated at home and in bars with friends and family to watch the match. This created a quite exceptional opportunity for residents of Bergamo to come together immediately after the first case had been reported in Lombardy and when, almost certainly, there were significant numbers of people who were infectious although asymptomatic (7). In addition, a recent report concluded that indoor transmission might have a larger impact than outdoor transmission in the diffusion of SARS-CoV-2, with sharing of indoor spaces being a major risk factor for the occurrence of the infection (8). Hence, this further underlines that gatherings, especially in public transports, in bars and clubs, and at home, might have had an important role in the diffusion of the disease at the local and regional level, probably with a greater impact than attendance of the match in the stadium.

So could this match explain what has happened subsequently in Bergamo? It is not the only football match to be implicated in the current pandemic. Public health staff have pointed to the match between Liverpool and Atletico Madrid, held in the Anfield stadium on 11th March, attended by 3,000 supporters from Madrid, the center of the pandemic in Spain. Others have questioned the wisdom of holding the Cheltenham horseracing festival, with races attracting crowds of over 60,000 people. SARS-CoV-2 hotspots have also been linked to parties and festivals, such as Mardi Gras in New Orleans, a part on Bondi Beach, and a carnival in Heinsberg, Germany, all events that have much in common with large sports events.

To answer this question we can look to the literature on outbreaks associated with mass gatherings. However, as one recent review notes (9), this almost entirely considers large religious gatherings, and especially the Hajj which brings over two million people from across the world to Mecca each year. A smaller number of reports describe outbreaks associated with festivals. In both, as might be expected, there have been a number of outbreaks of both gastrointestinal and respiratory infections, measles being the most commonly reported. There are, in contrast, many fewer reports of outbreaks associated with sporting events, again mostly clusters of measles. This conclusion is supported by an earlier systematic review examining reports of respiratory disease outbreaks associated with mass gatherings in the United States, which only found one arising from a sporting event (10). This was an outbreak of measles linked to a participant from abroad in a multi-day youth sporting event. There were no outbreaks associated with single day events. The authors emphasized the importance of close social contact as a risk factor for the transmission of airborne infections.

This paucity of reports is surprising, since those attending are often crowded together, with communal singing providing an important opportunity for exhaled viruses to spread. Thus, it is difficult to avoid the conclusion that transmission of airborne infectious agents at sporting events is substantially under-recognized.

Returning to Italian football, this time there has been an acceptance, even if hesitant, of the need to take action. Initially, Italian matches were played behind closed doors. However, it was not until 10th March that the decision to stop them completely was taken, when the number of COVID-19 cases and deaths in Italy were already 10,149, and 631, respectively (11). On the same day, the first football player in Italy tested positive for SARS-CoV-2.

Looking ahead, while countries differ in the details of their emerging plans to lift restrictions, most recognize that any change will have to be gradual, looking at the specifics of different types of gatherings (12). Given the scale of the financial interests involved, there will be tremendous pressure, not only from teams but also broadcasters and the many ancillary industries, to lift restrictions on football matches and other large sporting events such as horse racing or rugby as soon as there is any sign of the pandemic coming under control. Most politicians say they will be guided by the science but on this issue they face a challenge as the evidence is largely lacking. In these circumstances, it seems unwise to rush into lifting restrictions on these events and, when it happens, it should be accompanied by an intensive research effort to understand much better the mixing of people and, potentially, viruses that takes place in these circumstances, before, during, and after the matches.

Previous experience with mass gatherings held in Africa during the Ebola outbreak failed to find an association with increased transmission of the disease. However, this cannot be extrapolated to COVID-19 disease because of the different route of transmission and the high contagiousness of SARS-CoV-2 infection (13).

If sports competitions are resumed in the near future, we strongly believe that all matches should be held behind closed doors, paying special attention to gatherings in crowded places that could potentially occur in the immediate vicinity of stadiums. Should this not be possible, we believe that the use of face coverings as a means of source control, while not a substitute for social distancing which anyway cannot be maintained at large gatherings, should be made mandatory for spectators, given recent evidence supporting their role in reducing the transmission of the infection (14, 15). In addition, the implementation of intensified surveillance of those attending such events, at least for the immediate future, should be considered as a means of learning more about the dynamics of transmission of this disease and supporting tracking and containing infections (10, 16).

## Author Contributions

All authors contributed to creation and elaboration of the opinion article.

## Conflict of Interest

The authors declare that the research was conducted in the absence of any commercial or financial relationships that could be construed as a potential conflict of interest.

## References

[1] Ministero della Salute. Covid-19 – Situazione in Italia. (2020). Available online at: http://www.salute.gov.it/portale/nuovocoronavirus/dettaglioContenutiNuovoCoronavirus.jsp?area=nuovoCoronavirus&id=5351&lingua=italiano&menu=vuoto (accessed May 18, 2020).

[2] Istituto Nazionale di Statistica, Istituto Superiore di Sanità Impatto dell'epidemia covid-19 sulla mortalità totale della popolazione residente primo trimestre 2020. (2020). Available online at: https://www.istat.it/it/files/2020/05/Rapporto_Istat_ISS.pdf (accessed May 18, 2020).

[3] Ministero della Salute Covid-19: i casi in Italia alle ore 18 del 11 marzo. (2020). Available online at: http://www.salute.gov.it/portale/news/p3_2_1_1_1.jsp?lingua=italiano&menu=notizie&p=dalministero&id=4204 (accessed May 18, 2020).

[4] Ministero della Salute Covid-19, i casi in Italia alle ore 18 del 31 marzo. (2020). Available online at: http://www.salute.gov.it/portale/news/p3_2_1_1_1.jsp?lingua=italiano&menu=notizie&p=dalministero&id=4370 (accessed May 18, 2020).

[5] L'Eco di Bergamo Coronavirus, the real death toll: 4.500 victims in one month in the province of Bergamo. (2020). Available online at: https://www.ecodibergamo.it/stories/bergamo-citta/coronavirus-the-real-death-tool-4500-victims-in-one-month-in-the-province-of_1347414_11/ (accessed May 18, 2020).

[6] Boccia S Ricciardi W Joannidis J. What other countries can learn from Italy during the COVID-19 pandemic. JAMA Intern Med. (2020). 10.1001/jamainternmed.2020.1447. [Epub ahead of print]. 32259190

[7] Bai Y Yao L Wei T Tian F Jin DY Chen L. Presumed asymptomatic carrier transmission of COVID-19. JAMA. (2020) 323:1406–7. 10.1001/jama.2020.2565 32083643 PMC7042844

[8] Qian H Miao T Liu L Zheng X Luo D Li Y Indoor transmission of SARS-CoV-2. medRxiv [preprint]. (2020). 10.1101/2020.04.04.20053058 33131151

[9] Hoang VT Gautret P. Infectious diseases and mass gatherings. Curr Infect Dis Rep. (2018) 20:44. 10.1007/s11908-018-0650-9 30155747 PMC7088693

[10] Rainey JJ Phelps T Shi J. Mass gatherings and respiratory disease outbreaks in the United States – should we be worried? results from a systematic literature review and analysis of the national outbreak reporting system. PLoS ONE. (2016) 11:e0160378. 10.1371/journal.pone.0160378 27536770 PMC4990208

[11] Ministero della Salute Covid-19: i casi in Italia alle ore 18 del 10 marzo. (2020). Available online at: http://www.salute.gov.it/portale/news/p3_2_1_1_1.jsp?lingua=italiano&menu=notizie&p=dalministero&id=4192 (accessed May 18, 2020).

[12] European Commission Coronavirus: European roadmap shows path towards common lifting of containment measures. (2020). Available online at: https://ec.europa.eu/commission/presscorner/detail/en/IP_20_652 (accessed May 18, 2020).

[13] Zumla A McCloskey B Bin Saeed AA Dar O Al Otabi B Perlmann S. What is the experience from previous mass gathering events? Lessons for Zika virus and the Olympics 2016. Int J Infect Dis. (2016) 47:1–4. 10.1016/j.ijid.2016.06.010 27321962 PMC7110488

[14] Greenhalgh T Schmid MB Czypionka T Bassler D Gruer L. Face masks for the public during the covid-19 crisis. BMJ. (2020) 369:m1435. 10.1136/bmj.m1435 32273267

[15] Rodriguez-Palacios A Cominelli F Basson A Pizarro T Ilic S Textile masks and surface covers - a ‘universal droplet reduction model' against respiratory pandemics. medRxiv. (2020). 10.1101/2020.04.07.20045617 PMC7267001 32574342

[16] Thackway S Churches T Fizzell J Muscatello D Armstrong P. Should cities hosting mass gatherings invest in public health surveillance and planning? Reflections from a decade of mass gatherings in Sydney, Australia. BMC Public Health. (2009) 9:324. 10.1186/1471-2458-9-324 19735577 PMC2754454

